# Weight, Anthropometric and Metabolic Changes After Discontinuing Antiretroviral Therapy Containing Tenofovir Alafenamide in People With HIV

**DOI:** 10.1093/cid/ciae189

**Published:** 2024-04-12

**Authors:** José Damas, Aline Munting, Jacques Fellay, David Haerry, Catia Marzolini, Philip E Tarr, Ana Steffen, Dominique L Braun, Marcel Stoeckle, Enos Bernasconi, Olivier Nawej Tshikung, Christoph A Fux, Katharine E A Darling, Charles Béguelin, Gilles Wandeler, Matthias Cavassini, Bernard Surial, the Swiss HIV Cohort Study (SHCS), I Abela, K Aebi-Popp, A Anagnostopoulos, M Battegay, E Bernasconi, D L Braun, H C Bucher, A Calmy, M Cavassini, A Ciuffi, G Dollenmaier, M Egger, L Elzi, J Fehr, J Fellay, H Furrer, C A Fux, H F Günthard, A Hachfeld, D Haerry, B Hasse, H H Hirsch, M Hoffmann, I Hösli, M Huber, D Jackson-Perry, C R Kahlert, L Kaiser, O Keiser, T Klimkait, R D Kouyos, H Kovari, K Kusejko, N Labhardt, K Leuzinger, B Martinez de Tejada, C Marzolini, K J Metzner, N Müller, J Nemeth, D Nicca, J Notter, P Paioni, G Pantaleo, M Perreau, A Rauch, L Salazar-Vizcaya, P Schmid, R Speck, M Stöckle, P Tarr, A Trkola, G Wandeler, M Weisser, S Yerly

**Affiliations:** Infectious Diseases Service, University Hospital Lausanne, University of Lausanne, Lausanne, Switzerland; Infectious Diseases Service, University Hospital Lausanne, University of Lausanne, Lausanne, Switzerland; School of Life Sciences, École Polytechnique Fédérale de Lausanne, Lausanne, Switzerland; Biomedical Data Science Center, University Hospital and University of Lausanne, Lausanne, Switzerland; Chair of the Positive Council, Zurich, Switzerland; Division of Infectious Diseases and Hospital Epidemiology, University Hospital Basel, University Basel, Basel, Switzerland; Service of Clinical Pharmacology, University Hospital Lausanne, Lausanne, Switzerland; University Department of Medicine, Kantonsspital Bruderholz, University of Basel, Basel, Switzerland; Division of Infectious Diseases, Infection Prevention and Travel Medicine, Cantonal Hospital St Gallen, St. Gallen, Switzerland; Department of Infectious Diseases and Hospital Epidemiology, University Hospital Zurich, University of Zurich, Zurich, Switzerland; Division of Infectious Diseases and Hospital Epidemiology, University Hospital Basel, University Basel, Basel, Switzerland; Division of Infectious Diseases, Ente Ospedaliero Cantonale, Lugano, University of Geneva and University of Southern Switzerland, Lugano, Switzerland; Division of Infectious Diseases, University Hospital Geneva, University of Geneva, Geneva, Switzerland; Division of Infectious Diseases, Cantonal Hospital of Aarau, Aarau, Switzerland; Infectious Diseases Service, University Hospital Lausanne, University of Lausanne, Lausanne, Switzerland; Department of Infectious Diseases, Inselspital, Bern University Hospital, University of Bern, Bern, Switzerland; Department of Infectious Diseases, Inselspital, Bern University Hospital, University of Bern, Bern, Switzerland; Infectious Diseases Service, University Hospital Lausanne, University of Lausanne, Lausanne, Switzerland; Department of Infectious Diseases, Inselspital, Bern University Hospital, University of Bern, Bern, Switzerland

**Keywords:** antiretroviral therapy, tenofovir alafenamide, tenofovir disoproxil fumarate, weight, HIV

## Abstract

**Background:**

Antiretroviral therapy (ART)-related weight gain is of particular concern in people with HIV (PWH). Although weight gain was observed among PWH receiving tenofovir alafenamide (TAF), little is known about the potential reversibility after TAF discontinuation. We evaluated weight and metabolic changes 12 months after TAF discontinuation in the Swiss HIV Cohort Study.

**Methods:**

We included participants who received at least 6 months of TAF-containing ART between January 2016 and March 2023. Using multivariable mixed-effect models, changes in weight and lipid levels were compared between individuals who continued TAF and those who switched to one of the following TAF-free regimens: (1) tenofovir disoproxil fumarate (TDF)-based ART, (2) dolutegravir/lamivudine (DTG/3TC), or (3) long-acting cabotegravir/rilpivirine (CAB/RPV).

**Results:**

Of 6555 participants (median age 54 years, 24.3% female, 13% Black), 5485 (83.7%) continued, and 1070 (16.3%) stopped TAF. Overall, discontinuing TAF was associated with an adjusted mean weight change of −0.54 kg (95% confidence interval [CI] −.98 to −.11) after 12 months. In stratified analyses, switching from TAF to TDF led to an adjusted mean weight decrease of −1.84 kg (95% CI −2.72 to −.97), and to a decrease in mean total cholesterol (−0.44 mmol/L) and triglycerides (−0.38 mmol/L) after 12 months. Switching from TAF-based ART to DTG/3TC (−0.17 kg, 95% CI −.82 to .48) or long-acting CAB/RPV (−0.64 kg, 95% CI −2.16 to .89) did not lead to reductions in weight.

**Conclusions:**

Replacing TAF with TDF in PWH led to a decrease in body weight and an improved lipid profile within 1 year. Weight changes were not observed among individuals who switched to DTG/3TC or long-acting CAB/RPV.

## Teaser

In the Swiss HIV Cohort Study, replacing tenofovir alafenamide (TAF) with tenofovir disoproxil fumarate (TDF) resulted in a −1.84 kg weight decrease within 1 year. No substantial weight changes were noted after switching from TAF to dolutegravir/lamivudine or cabotegravir/rilpivirine.

## Graphical Abstract

**Graphical Abstract:**
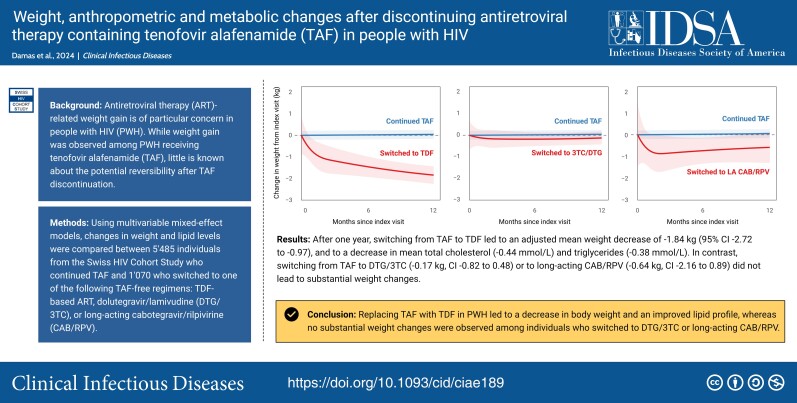
This graphical abstract is also available at Tidbit: https://tidbitapp.io/tidbits/weight-anthropometric-and-metabolic-changes-after-discontinuing-antiretroviral-therapy-containing-tenofovir-alafenamide-taf-in-people-with-hiv-9af4bba9-2a15-4648-9696-d7c45ea94bcd

**(See the Editorial Commentary by Hill on pages 1006–9; Review Article by Wohl on pages 999–1005.)**

As life expectancy of people with HIV (PWH) is approaching that of the general population, the management and prevention of cardiometabolic conditions, including obesity and cardiovascular disease, have emerged as important issues in the care of PWH [1, 2]. Because the prevalence of obesity is increasing in PWH, antiretroviral therapy (ART)-related weight gain is of particular concern [3–6]. Studies have shown larger weight increases in treatment-naïve individuals starting tenofovir alafenamide (TAF) than in those starting tenofovir disoproxil fumarate (TDF), and weight gain after switching from TDF to TAF [7, 8]. However, little is known about weight and lipid profile trajectories after stopping TAF-based ART, and whether these metabolic changes are reversible.

The discontinuation of TAF did not lead to a decrease in weight in 2 randomized trials. In TANGO, no difference in weight changes was observed between individuals who switched away from TAF-based ART to dolutegravir and lamivudine (DTG/3TC), compared to those who remained on TAF [9]. Similarly, weight trajectories in PWH who switched from bictegravir/TAF/FTC to long-acting cabotegravir (CAB)/rilpivirine (RPV) were similar to those of people continuing bictegravir/TAF/FTC in the SOLAR study [10]. In contrast, replacing TAF with TDF let to a decrease in weight among 70 PWH in South Africa, and to improvements in lipid profiles among 146 individuals in Finland, indicating some potential for metabolic improvements after ART modification [11, 12].

The 2 clinical trials and the observational studies are based on small numbers of participants, which limits their generalizability. Studies from large and well-described cohorts have the potential to inform evidence-based shared decision-making with persons who become overweight or obese on ART. Therefore, we used data from the nationwide Swiss HIV Cohort Study (SHCS) to evaluate weight and metabolic changes after stopping TAF-based ART and adopting 1 of 4 TAF-free strategies: (1) replacing TAF with TDF, (2) switching from TAF-based ART to DTG/3TC, (3) switching to long-acting CAB/RPV, or (4) using other TAF-free ART.

## METHODS

### Study Design

We used data from the SHCS (www.shcs.ch), a prospective cohort established in 1988, which includes close to 80% of PWH who receive ART in Switzerland, and who are followed in 1 of 5 University Hospitals, 2 regional hospitals, 15 affiliated hospitals or by a registered private physicians [13]. Sociodemographic, clinical, laboratory, and behavioural data are prospectively recorded at registration and every 6 months thereafter using standardized protocols (www.shcs.ch/292-instruction). Assessments at every follow-up visit include weight and anthropometric measurements, documentation of all changes in medication (including ART and comedication), as well as glucose and lipid measurements. Data quality and consistency are ensured by quality checks and regular site visits of participating centers. All centers’ local ethical committees approved the cohort study, and all participants provided written informed consent. The reporting of the study follows the Strengthening the Reporting of Observational Studies in Epidemiology (STROBE) guidelines [14].

### Eligibility Criteria

We considered cohort participants who received at least 6 months of TAF-containing ART between 1 January 2016, the year in which TAF was approved in Switzerland, and 31 March 2023 (database closure). The index visit was defined as the date of treatment change for participants who discontinued TAF. For individuals who remained on TAF, the index visit was defined as the median date at which participants discontinued TAF (27 July 2021). Female participants who became pregnant during the study period, and participants with missing data on baseline covariables (weight, human immunodeficiency virus type 1 [HIV-1] viral load, CD4 cell count and smoking) were excluded. The follow-up for participants who discontinued the cohort after the index visit was censored at that time.

### Outcomes and Definitions

The primary aim was to compare weight trajectories over time between PWH who continued a TAF-based ART regimen and those who discontinued TAF, and to estimate the difference in weight between the index visit and 12 months thereafter. We performed separate analyses according to the ART regimen after stopping TAF. TAF-free ART regimens were classified into regimens containing tenofovir disoproxil (TDF), dual therapy with DTG/3TC, long-acting CAB/RPV, and other TAF-free ART combinations. To account for different changes in weight before the index visit, we included all weight measurements 2.5 years before the index visit until the end of each individual's follow-up. The main exposure of interest was discontinuation of TAF compared with continuing TAF. Secondary outcomes were changes in waist-to-hip ratio (WHR), and mean changes in total cholesterol, low-density lipoprotein (LDL) cholesterol, high density lipoprotein (HDL) cholesterol, triglycerides, and total cholesterol-to-HDL ratio. Weight categories were classified according to body mass index (BMI) as underweight (<18.5 kg/m^2^), normal (18.5–24.9 kg/m^2^), overweight (25–29.9 kg/m^2^), and obese (≥30.0 kg/m^2^) [15]. Diabetes at index visit was defined as hemoglobin A1c levels ≥6.5%, any glucose level above 11 mmol/L or treatment with antidiabetic medication. History of cardiovascular disease included myocardial infarction, stroke, and/or invasive cardiovascular procedures. The occurrence of both diabetes and cardiovascular events were reported by the treating cohort physician using standardized forms.

### Statistical Analysis

Patient characteristics between individuals who continued and those who discontinued TAF were compared using *x*^2^ and Wilcoxon rank-sum tests. Adjusted mean changes in weight over time in absolute values were estimated using multivariable mixed-effect models, with random intercepts for each individual. In order to allow nonlinear weight trajectories, time was modeled using restricted cubic splines with 3 knots. Covariates were pre-specified characteristics that are associated with weight changes, the decision to discontinue a TAF containing regimen, or both. Multivariable analyses for mean weight changes over time were adjusted for time-fixed values of age, sex, Black people, CD4 cell count at the index visit (cells/mm^3^), years on ART, and weight at the index visit (in kilograms), and for time-varying variables, including the use of integrase inhibitors (INSTI, yes/no), physical activity (exercising more than twice a week, 1–2 times per week, 1–4 times per month, never or unknown), smoking (yes/no), and use of comedication associated with weight changes (including antidepressants, neuroleptic drugs, corticosteroids, and antidiabetic drugs) [16].

Mean differences in serum lipid levels were estimated similarly to weight analyses using the same random-effects structure and were adjusted for age, sex, Black people, and individual lipid level at the index visit, as well as for the time-varying values of weight, physical activity, and the use of lipid-lowering drugs (statins, fibrates, and nicotinic acid). Observations with missing values in variables of interest were censored (see Supplementary appendix figure 1), except for physical activity where missing values were added as a category (unknown). All statistical analyses were performed using R, version 4.2.3.

### Subgroup and Sensitivity Analyses

As female PWH and Black people are at particular risk for weight increases on TAF-based ART, we performed pre-specified subgroup analyses in male and female, and in Black people and non-Black people using interaction terms [7, 8]. We repeated the main analysis among individuals with excessive weight gain, defined as an increase of more than 10% of body weight from TAF start until the index visit.

Most participants who switched from TAF to TDF also discontinued INSTI at the same time. To understand whether the changes in weight were driven by the switch to TDF or the discontinuation of an INSTI, we performed a post-hoc analysis of weight changes after switching to TDF in the subset of individuals who did not receive an INSTI until the index visit.

### Patient and Public Involvement

A patient and public involvement (PPI) representative from the Swiss HIV Cohort Study (http://www.shcs.ch/315-patient-and-public-involvement-ppi) was involved in the study design, data analysis and critical review of the manuscript, and is included as co-author.

## RESULTS

### Study Population

Of 11 782 participants under follow-up between 1 January 2016 and 31 March 2023, 7685 received a TAF-containing ART regimen for more than 6 months. We excluded 995 individuals without any follow-up after the index visit, 59 pregnant females, and 76 participants with missing information on covariates at the index visit (Supplementary Figure 1). The final study population included 6555 participants. The median age was 54 years (interquartile range [IQR], 45.0 to 60.0 years), 24.3% were female, 13.3% were Black people, and 95.1% had a suppressed HIV viral load at the index visit. The median weight at the index visit was 76.0 kg (IQR, 67.0 to 87.0 kg), and 50.2% were overweight or obese.

Of the 6555 participants, 5485 (83.7%) continued TAF until the end of the study, and 1070 (16.3%) switched to a TAF-free regimen. Of the individuals who discontinued TAF, 196 (18.3%) participants started a TDF-containing regimen, 565 (52.8%) switched to 3TC/DTG, 115 (10.7%) started long-acting CAB/RPV, and 194 (18.1%) switched to other TAF-free ART combinations. Among individuals who switched from TAF to TDF, the most common ART regimens used were doravirine/TDF/3TC (n = 642, 60.1%), followed by RPV/TDF/FTC (n = 147, 13.8%). Compared to individuals who continued TAF, those who stopped the drug were younger, had received ART for a shorter duration, were more likely to be overweight or obese, and were less likely to have diabetes or a history of cardiovascular disease. In addition, total cholesterol and LDL levels were slightly higher among individuals who discontinued TAF than in those who continued the drug, while total cholesterol-HDL ratios were similar in both groups (Table 1). The median follow-up after the index date was 1.3 years (IQR 1.1 to 1.4) for individuals continuing TAF, and 1.2 years (IQR 0.6 to 2.3) for those who stopped TAF.

**Table 1.** Characteristics of the Study Population at the Index Visit

Characteristic	Continued TAF (N = 5485)	Stopped TAF (N = 1070)	*P V*alue
Median age, years (IQR)	54.0 (45.0 to 60.0)	50.0 (41.0 to 58.0)	<.001
Female, n (%)	1347 (24.6)	243 (22.7)	.21
HIV transmission group, n (%)			<.001
Heterosexual female	1035 (18.9)	193 (18.0)	
Heterosexual male	906 (16.5)	170 (15.9)	
Women who inject drugs	115 (2.1)	16 (1.5)	
Men who inject drugs	236 (4.3)	18 (1.7)	
Men having sex with men	2728 (49.7)	593 (55.4)	
Other female	65 (1.2)	7 (0.7)	
Other male	118 (2.2)	11 (1.0)	
*Missing*	282 (5.1)	62 (5.8)	
Black people, n (%)	724 (13.2)	131 (12.2)	.42
Median duration of ART before index visit, years (IQR)	14.5 (8.6 to 23.1)	10.3 (5.4 to 16.7)	<.001
Median duration of TAF-containing ART before index visit, years (IQR)	4.3 (3.1 to 5.2)	4.8 (3.8 to 5.4)	<.001
Median CD4 count, cells/μL (IQR)	670 (497 to 874)	704 (541 to 885)	.001
Median CD4 nadir, cells/μL (IQR)	214 (106 to 338)	264 (156 to 376)	<.001
HIV-1 RNA viral load <50 copies/mL, n (%)	5183 (94.5)	1044 (97.6)	<.001
On INSTI-containing ART, n (%)	4139 (75.5)	788 (73.6)	.19
Bictegravir	2579 (47.0)	278 (26.0)	
Dolutegravir	661 (12.1)	243 (22.7)	
Elvitegravir	732 (13.3)	247 (23.1)	
Raltegravir	167 (3.0)	20 (1.9)	
Self-reported adherence, n (%)			.32
Missed 1 dose or more per week	258 (4.7)	43 (4.1)	
Missed 1 dose per month	464 (8.5)	85 (7.9)	
Never missed a dose	4743 (86.5)	941 (87.9)	
*Missing*	20 (0.4)	1 (0.1)	
Use of weight-modifying drugs, n (%)	549 (10.0)	85 (7.9)	.06
Median weight, kg (IQR)	75 (66 to 86)	79 (69 to 89)	<.001
Median BMI, kg/m^2^ (IQR)	24.9 (22.4 to 27.8)	25.6 (23.2 to 28.9)	<.001
BMI category, n (%)			<.001
Underweight (<18.5 kg/m^2^)	202 (3.7)	21 (2.0)	
Normal (18.5–24.9 kg/m^2^)	2611 (47.6)	427 (39.9)	
Overweight (25.0–29.9 kg/m^2^)	1875 (34.2)	413 (38.6)	
Obese (≥30.0 kg/m^2^)	797 (14.5)	209 (19.5)	
Diabetes, n (%)	500 (9.1)	63 (5.9)	.001
History of CVD, n (%)	501 (9.1)	68 (6.4)	.004
Median eGFR, mL/min (IQR)	84.9 (71.8 to 98.2)	87.3 (74.7 to 100.8)	<.001
Current smoker, n (%)	1882 (34.3)	299 (27.9)	<.001
Median total cholesterol (IQR)			<.001
mmol/L	4.9 (4.2 to 5.6)	5.00 (4.3 to 5.8)	
mg/dL	187.6 (161.6 to 216.6)	193.4 (166.3 to 224.3)	
Median HDL cholesterol (IQR)			.40
mmol/L	1.30 (1.1 to 1.6)	1.30 (1.1 to 1.6)	
mg/dL	49.9 (40.6 to 60.3)	50.3 (41.4 to 61.1)	
Median LDL cholesterol (IQR)			<.001
mmol/L	2.7 (2.1 to 3.4)	2.9 (2.2 to 3.5)	
mg/dL	104.8 (80.3 to 130.1)	109.7 (84.8 to 135.8)	
Median triglyceride cholesterol (IQR)			.45
mmol/L	1.5 (1.0 to 2.2)	1.4 (1.0 to 2.1)	
mg/dL	131.1 (88.6 to 196.6)	127.9 (88.6 to 188.7)	
Total cholesterol-HDL ratio (IQR)	3.7 (3.0 to 4.6)	3.8 (3.1 to 4.8)	.05
Receiving lipid-lowering therapy, n (%)	1322 (24.1)	192 (17.9)	<.001

Abbreviations: ART, antiretroviral therapy; BMI, body mass index; CVD, cardiovascular disease/events; eGFR, estimated glomerular filtration rate; HIV-1, human immunodeficiency virus type 1; HDL, high-density lipoprotein; IQR, interquartile range; LDL, low-density lipoprotein; TAF, tenofovir alafenamide.

### Changes in Weight

**Figure 1.**
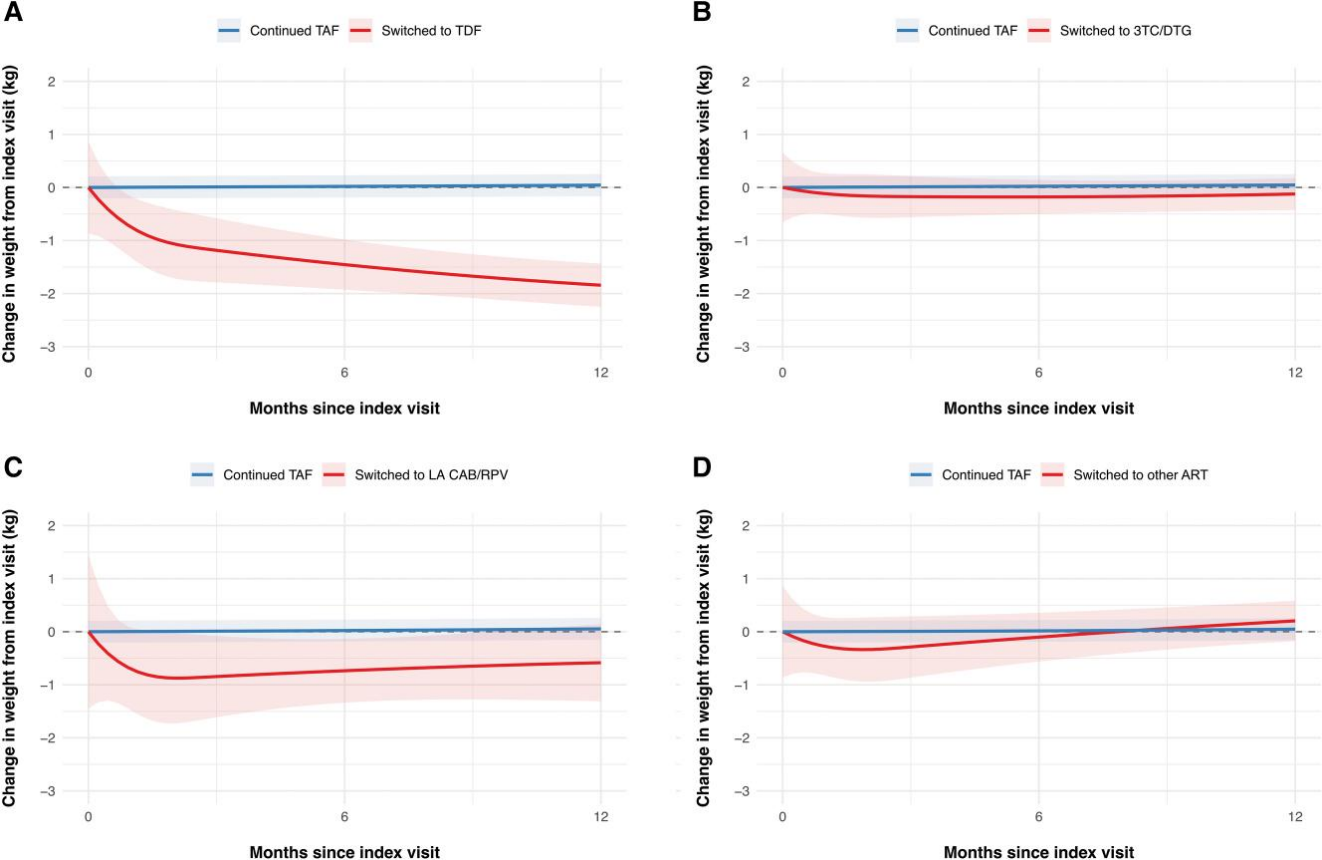
Weight changes over time after index visit, stratified by the TAF-free ART regimen. Mean changes in weight (line) and corresponding 95% CIs (shaded area) of continuing TAF-based ART (*blue line*) compared with (*A*) switching to TDF-based ART, (*B*) switching to DTG/3TC, (*C*) switching to LA CAB/RPV, or (*D*) switching to other ART (*red lines*). All models were adjusted for age, sex, ethnicity, CD4-cell count, use of integrase inhibitors, physical activity, smoking status, and use of weight-modifying drugs. The model includes random intercepts for each individual. A total of 6282 PWH were included in the analyses. Abbreviations: ART, antiretroviral therapy; CAB/RPV, cabotegravir/rilpivirine; CI, confidence interval; DTG/3TC, dolutegravir/lamivudine; LA, long-acting; PWH, people with human immunodeficiency virus; TAF, tenofovir alafenamide; TDF, tenofovir disoproxil fumarate.

In unadjusted analyses, participants who discontinued TAF experienced a mean weight change of −0.24 kg (95% CI, −.68 to .19) in the first 12 months after the index visit, compared with a weight change of 0.04 kg (95% CI, −.03 to .11) in individuals with continuous use of TAF (between group difference −0.28 kg, 95% CI −.72 to .16). After adjusting for confounders, discontinuing TAF was associated with a mean weight decrease of −0.54 kg (95% CI, −.98 to −.11) 12 months after the index visit, compared with 0.03 kg (95% CI, −.03 to .10) with the continuous use of TAF (between group difference −0.58 kg, 95% CI, −1.02 to −.14, Supplementary Figure 2, Table 2). Decreases in weight after stopping TAF were only observed in individuals who switched to a TDF-containing regimen (mean adjusted weight change after 12 months −1.89 kg, 95% CI −2.76 to −1.01), whereas no changes in weight were observed in individuals who switched to a DTG/3TC, long-acting CAB/RPV or other ART regimens without TAF (Figure 1, Table 2).

**Table 2.** Adjusted Changes in Weight From The Index Visit to 12 Months Thereafter, in the Overall Study Population and Across Subgroups^a^

Variable	Continued TAF, kg (95% CI)	Stopped TAF, kg (95% CI)	Difference Between the 2 Groups	*P V*alue for Difference
Overall	0.03 (−.03 to .10)	−0.54 (−.98 to −.11)	−0.58 (−1.02 to −0.14)	.010
Sex				
Female	−0.06 (−.20 to .07)	−0.89 (−1.69 to −.09)	−0.83 (−1.64 to −0.10)	.046
Male	0.07 (−.01 to .14)	−0.43 (−.94 to .09)	−0.49 (−1.01 to 0.02)	.06
Race				
Black people	−0.05 (−.24 to .14)	−1.24 (−2.40 to −.08)	−1.19 (−2.37 to −0.01)	.048
Non-Black people	0.05 (−.02 to .12)	−0.43 (−.90 to .03)	−0.48 (−0.95 to −0.01)	.046
Subgroup analyses according to TAF-free regimen^b^				
Switched to TDF	0.05 (−.02 to .11)	−1.84 (−2.72 to −.97)	−1.89 (−2.76 to −1.01)	<.001
Switched to 3TC/DTG	0.05 (−.02 to .11)	−0.12 (−.77 to .53)	−0.17 (−0.82 to 0.48)	.61
Switched to CAB/RPV	0.05 (−.02 to .11)	−0.58 (−2.11 to .94)	−0.64 (−2.16 to 0.89)	.41
Other	0.05 (−.02 to .11)	0.20 (−.66 to 1.07)	0.16 (−0.71 to 1.02)	.72

Abbreviations: CAB/RPV, cabotegravir/rilpivirine; CI, confidence interval; 3TC/DTG, lamivudine/dolutegravir; kg, kilograms; TAF, tenofovir alafenamide; TDF, tenofovir disoproxil fumarate.

^a^Adjusted for the time fixed covariates age, sex, Black people, weight at index visit, use of integrase strand transfer inhibitor, smoking status, time in antiretroviral therapy, CD4 cell count at index visit, and time-varying physical activity, use of weight-modifying drugs, and human immunodeficiency virus type 1 (HIV-1) viral load. Models include random intercepts for each patient.

^b^Only applies to the individuals who stopped TAF. The comparator group of individuals who continued TAF remained unchanged. Minor discrepancies in estimates from this group and the overall group are due to the model estimation process.

In subgroup analyses, the decrease in weight was more pronounced among female participants (*P* value <.001 for the interaction with sex, Supplementary Figure 2), and among Black people than non-Black people (*P* < .001 for the interaction with ethnicity, Table 2). In the subgroup of individuals who experienced a weight gain of more than 10% after starting TAF, individuals who stopped TAF decreased their weight 12 months after index visit (−1.76 kg, 95% CI −3.12 to −.41), however, a decrease was also observed in individuals who continued TAF (−0.44 kg, 95% CI −.67 to −.21, between-group difference −1.32 kg, 95% CI −2.70 to .06). In individuals who did not receive an INSTI until the index visit (n = 65), replacing TAF with TDF was associated with a decrease of −1.03 kg (95% CI −2.35 to .29).

### Changes in Lipid Levels and Waist-to-Hip Ratio

**Figure 2.**
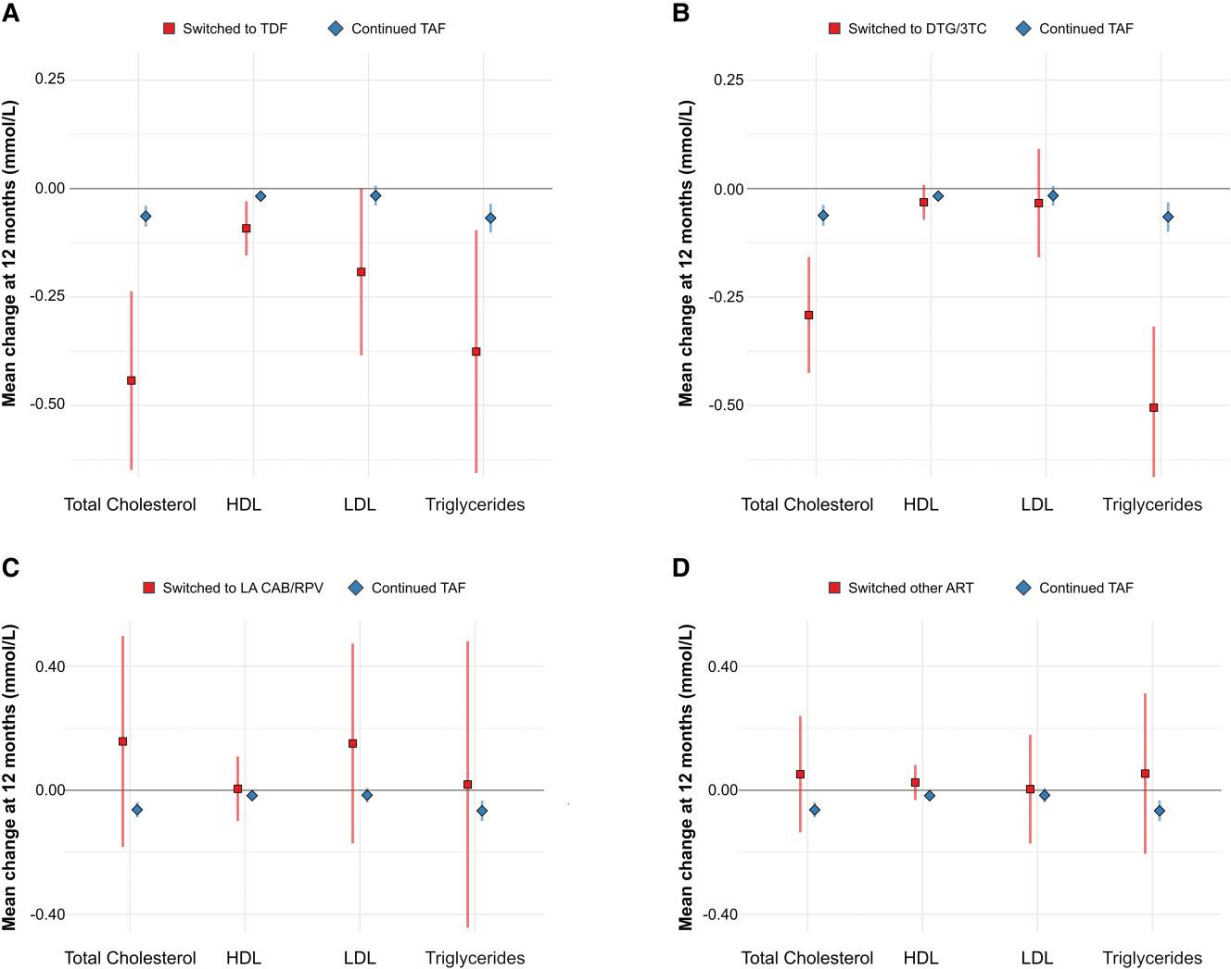
Changes in lipid levels 12 m after the index visit, stratified by the TAF-free ART regimen. Mean changes in lipid levels (*squares and diamonds*) and corresponding 95% CIs (*vertical lines*) after 12 m of continuing TAF-based ART (*diamonds and lines in blue*) compared with (*A*) switching to TDF-based ART, (*B*) switching to DTG/3TC, (*C*) switching to LA CAB/RPV, or (*D*) switching to other ART (*squares and lines in red*). All models were adjusted for age, sex, ethnicity, individual lipid level at baseline, and time-varying physical activity, weight, and use of lipid-lowering drugs. The model includes random intercepts for each individual. Abbreviations: ART, antiretroviral therapy; CAB/RPV, cabotegravir/rilpivirine; CI, confidence interval; DTG/3TC, dolutegravir/lamivudine; HDL, high-density lipoprotein; LA, long-acting; LDL, low-density lipoprotein; TAF, tenofovir alafenamide; TDF, tenofovir disoproxil fumarate.

In adjusted analyses, discontinuing TAF was associated with a decrease in total cholesterol, triglycerides and total cholesterol-HDL ratio (Table 3, Supplementary Figure 3). These changes were most pronounced in individuals who switched from TAF to either TDF or 3TC/DTG (Figure 2, Supplementary Tables 1–4). During follow-up, 172 (3.1%) of PWH continuing TAF-based regiments started a lipid-lowering drug versus 38 (3.6%) who discontinued TAF (−0.4 percentage points, 95% CI −1.7 to .8).

**Table 3.** Adjusted Mean Differences of Lipid Levels After 12 Months Compared With Index Visit After Discontinuing TAF

Variable	Continued TAF (95% CI)	Stopped TAF (95% CI)	Difference Between the Two Groups	*P V*alue for Difference
Total cholesterol				.005
mmol/L	−0.06 (−.09 to −.04)	−0.20 (−.29 to −.11)	−0.14 (−0.23 to −0.04)	
mg/dL	−2.5 (−3.4 to −1.5)	−7.7 (−11.2 to −4.1)	−5.2 (−8.9 to −1.6)	
HDL cholesterol				.543
mmol/L	−0.02 (−.02 to −.01)	−0.03 (−.05 to .001)	−0.01 (−0.04 to 0.02)	
mg/dL	−0.07 (−.9 to −.4)	−1.0 (−2.1 to .1)	−0.3 (−1.5 to 0.8)	
LDL cholesterol				.644
mmol/L	−0.02 (−.04 to .001)	−0.04 (−.12 to .05)	−0.02 (−0.11 to 0.07)	
mg/dL	−0.7 (−1.5 to .2)	−1.5 (−4.8 to 1.5)	−0.8 (−4.2 to 2.6)	
Triglycerides				<.001
mmol/L	−0.07 (−.10 to −.03)	−0.30 (−.43 to −.17)	−0.23 (−0.36 to −0.10)	
mg/dL	−5.9 (−8.9 to −2.9)	−26.2 (−37.8 to −14.7)	−20.4 (−32.3 to −8.5)	
Total cholesterol-HDL ratio	−0.02 (−.06 to .02)	−0.18 (−.33 to −.03)	−0.16 (−0.31 to −0.01)	.036

Models adjusted for time fixed covariables age, sex, ethnicity, individual lipid level at baseline: and time-varying physical activity, weight, and use of lipid-lowering drugs.

Abbreviations: CI, confidence interval; HDL, high-density lipoprotein; IQR, interquartile range; LDL, low-density lipoprotein; TAF, tenofovir alafenamide.

Twelve months after index visit, the mean change in WHR was −0.0049 (95% CI −.0115 to .0017) in individuals who discontinued TAF, compared with 0.0021 (95% CI, .0010 to .0032) in those with continuous use of TAF (between group difference −0.0070, 95% CI, −.0137 to −.0003). The difference in WHR between individuals who stopped TAF and those who continued a TAF-containing regimen was larger in male than in female participants (0.0084, 95% CI .0004 to .0164 compared to 0.0033, 95% CI −.0090 to .0156, Supplementary Figure 4).

## DISCUSSION

In this nationwide cohort study, replacing TAF with TDF led to a decrease in weight and small changes in WHR, whereas no substantial weight changes were noted among individuals who switched to other TAF-free regimens such as CAB/RPV or 3TC/DTG. Changes in weight among individuals who switched to TDF were followed by a decrease in total cholesterol and triglycerides levels. Similar changes in lipid levels were found among PWH switching from TAF to 3TC/DTG.

Our findings of a decrease in weight after switching from TAF- to TDF-based ART are in line with those from the CHARACTERISE study [12], a sub-study from the ADVANCE trial assessing weight trajectories from 172 PWH following a series of ART changes [8]. In CHARACTERISE, female PWH who switched from DTG/TAF/FTC to DTG/TDF/3TC lost 1.6 kg after 192 weeks of follow-up, whereas no change was observed in men [12]. In the present study, weight decreases were only observed in individuals who had TAF replaced by TDF but not in those who switched to other TAF-free ART regimens. These findings together with those from the CHARACTERISE study provide evidence for a weight-suppressing effect of TDF, rather than a weight-promoting impact of TAF. Additional evidence for this hypothesis has been gathered in HIV pre-exposure prophylaxis (PrEP) studies, where participants receiving TDF/FTC were more likely to experience a loss of at least 5% of body weight compared with individuals receiving placebo or CAB [17, 18]. In another study from the ATHENA Cohort, weight trajectories from 115 PWH who experienced excessive weight gain on TAF or INSTI-based ART were evaluated after discontinuing TAF, INSTI, or both. After 12 months of follow-up, weight loss was most notable among individuals who discontinued both TAF and INSTI (−2.6 kg, 95% CI −5.8 to .02), followed by individuals who discontinued TAF (−1.9 kg, 95% CI −3.4 to −.4) or INSTI alone (−1.9 kg, 95% CI −3.9 to .1) [19]. However, a sensitivity analysis in the same study did not find an association of weight loss after switching to TDF [19].

We found no differences in weight when TAF-based regimens were switched to either DTG/3TC or CAB/RPV, two common TAF-free ART regimens in clinical practice in Switzerland. These results confirm and extend the findings from the randomized trials TANGO and SOLAR. In the TANGO trial, 369 PWH who received TAF experienced a weight increase of 2.2 kg after being switched to 3TC/DTG, whereas 371 individuals who remained on TAF gained 1.7 kg (between-group differences 0.49 kg, 95% CI, −.46 to 1.44) [9]. In the SOLAR trial, no weight change was observed between 277 PWH who continued BIC/TAF/FTC and 454 who switched to long-acting CAB/RPV [10]. Weight loss associated with a switch from TAF to TDF suggests a weight suppressive effect of TDF [20]. However, in our study, most individuals who switched from TAF to TDF also switched from an INSTI-based to a NNRTI-based ART regimen: 61% switched to doravirine/TDF/3TC, and 13% to RPV/TDF/FTC; with only 12% continuing INSTI-based therapy. The absence of INSTI in most TDF-based regiments may have contributed in part to the reduction of weight that we observed in this subgroup, as INSTI are also associated with weight increase. However, in a post hoc analysis restricted to individuals who did not receive INSTI-based ART at the index visit, switching from TAF to TDF remained associated with a decrease in weight, indicating that these changes cannot be attributed to the impact of discontinuing INSTI alone.

We observed a decrease of lipid levels after TAF discontinuation. These changes were also mainly driven by participants who switched to TDF-based regimens, which was anticipated as TDF has a favourable impact on lipid profiles [11, 21]. In addition, improvements were observed among individuals who had their TAF-based regimen replaced by DTG/3TC. These results align well with the findings of the TANGO study, in which participants assigned to the DTG/3TC arm experienced both a decrease in total cholesterol and triglycerides compared to those assigned to the TAF-based arm [9]. Similarly, an observational study from Spain found a decrease in cholesterol levels among 118 PWH who switched from TAF-based ART to either DTG/3TC or DTG/RPV [22].

Our study is among the largest to date to evaluate the impact of discontinuing TAF on weight and other metabolic outcomes in PWH. The rich data of the Swiss HIV Cohort Study allowed us to adjust our analyses for a wide range of confounders (including physical activity and weight modifying drugs), and to perform subgroup analyses of clinical interest. However, the follow-up period of 1 year was relatively short, which limits our ability to grasp the full effect of switching to a TAF-free regimen on longer term weight trajectories. Specifically, CAB/RPV was licensed in Switzerland in March 2022 only, and the analysis of individuals who switched from TAF-based ART to CAB/RPV was based on a small number of individuals with relatively short follow-up. Therefore, we may have been underpowered to detect a potential difference in weight changes between individuals who switched to CAB/RPV and those who remained on TAF. In addition, as only 24% of our study population were female participants, and 13% were Black people, the generalizability of these subgroup analyses remains limited, and confirmation in other studies is warranted. Although we accounted for many confounders including weight trajectories prior to the index visit, some residual confounding by indication could remain, as participants who stopped TAF had a higher weight at baseline compared with individuals who remained on TAF.

In conclusion, replacing TAF with TDF in PWH led to a decrease in body weight, whereas switching to other common TAF-free regimens such as DTG/3TC or CAB/RPV did not lead to substantial weight changes. These findings provide evidence to guide shared decision-making with patients affected by ART-induced overweight or obesity. The potential weight and metabolic benefits of replacing TAF with TDF must be weighed against the safety profile of TAF, which includes improvements in renal function among individuals with chronic kidney disease, and lower rates of bone demineralization [23–26]. Further studies are needed to assess whether these weight changes are sustained over time, and whether they have an impact on the incidence of weight-related cardiometabolic comorbidities among PWH.

## Supplementary Data

Supplementary materials are available at *Clinical Infectious Diseases* online. Consisting of data provided by the authors to benefit the reader, the posted materials are not copyedited and are the sole responsibility of the authors, so questions or comments should be addressed to the corresponding author.

## Supplementary Material

ciae189_Supplementary_Data

## Notes

***Author contributions.*** J. D., B. S., M. C., and G. W. conceived and designed the study. J. D. and B. S. performed the statistical analyses. J. D. and B. S. drafted the initial manuscript. All authors contributed data to the study and to the interpretation of the results and revised the manuscript for substantial content. All authors read and approved the final manuscript.

***Acknowledgments.*** The authors thank all participants, physicians, and nurses associated with the Swiss HIV Cohort Study (SHCS). The members of the SHCS are I. Abela, K. Aebi-Popp, A. Anagnostopoulos, M. Battegay, E. Bernasconi, D.L. Braun, H.C. Bucher, A. Calmy, M. Cavassini, A. Ciuffi, G. Dollenmaier, M. Egger, L. Elzi, J. Fehr, J. Fellay, H. Furrer, C.A. Fux, H.F. Günthard (President of the SHCS), A. Hachfeld, D. Haerry (deputy of “Positive Council”), B. Hasse, H.H. Hirsch, M. Hoffmann, I. Hösli, M. Huber, D. Jackson-Perry (patient representative), C.R. Kahlert (Chairman of the Mother & Child Substudy), L. Kaiser, O. Keiser, T. Klimkait, R.D. Kouyos, H. Kovari, K. Kusejko (Head of Data Center), N. Labhardt, K. Leuzinger, B. Martinez de Tejada, C. Marzolini, K.J. Metzner, N. Müller, J. Nemeth, D. Nicca, J. Notter, P. Paioni, G. Pantaleo, M. Perreau, A. Rauch. (Chairman of the Scientific Board), L. Salazar-Vizcaya, P. Schmid, R. Speck, M. Stöckle (Chairman of the Clinical and Laboratory Committee), P. Tarr, A. Trkola, G. Wandeler, M. Weisser, S. Yerly.

***Financial support*.** This work was funded within the framework of the Swiss HIV Cohort Study, supported by the Swiss National Science Foundation (SNF grant number 201369, SHCS project number 894), and by the SHCS research foundation. Data are gathered by the Five Swiss University Hospitals, 2 Cantonal Hospitals, 15 affiliated hospitals, and 36 private physicians (listed at www.shcs.ch/180-health-care-providers).
