# Supplementary material for: Weight, Anthropometric and Metabolic Changes After Discontinuing Antiretroviral Therapy Containing Tenofovir Alafenamide in People With HIV
Source: Clin Infect Dis. 2024 Apr 12;79(4):990–8. doi: 10.1093/cid/ciae189 (PMC11478808; doi:10.1093/cid/ciae189)
Supplement: ciae189_Supplementary_Data [file ciae189_supplementary_data.docx]

**Supplementary Material**

## Weight, anthropometric and metabolic changes after discontinuing antiretroviral therapy containing tenofovir alafenamide (TAF) in people with HIV

José **Damas**, Aline **Munting**, Jacques **Fellay**, David **Haerry**, Catia **Marzolini**, Philip E. **Tarr**, Ana **Steffen***,* Dominique L. **Braun**, Marcel **Stoeckle**, Enos **Bernasconi**, Olivier **Nawej Tshikung**, Christoph A. **Fux**, Katharine E.A. **Darling**, Charles **Béguelin**, Gilles **Wandeler**, Matthias **Cavassini**, Bernard **Surial**, *and the* ***Swiss HIV Cohort Study*** *(SHCS)*

**Table of contents:**

**Figure S1:** Selection of the study population 2

**Figure S2:** Changes in weight over time overall and stratified by sex 3

**Figure S3:** Adjusted mean changes lipid levels overall 4

**Figure S4:** Changes in waist-to-hip ratio overall and stratified by sex 5

**Table S1:** Adjusted mean differences in lipid levels (switching from TAF to TDF) 6

**Table S1:** Adjusted mean differences in lipid levels (switching from TAF to DTG/3TC) 7

**Table S1:** Adjusted mean differences in lipid levels (switching from TAF to CAB/RPV) 8

**Table S1:** Adjusted mean differences in lipid levels (switching from TAF to other ART) 9

***Figure S1.*** Selection of the study population.

**
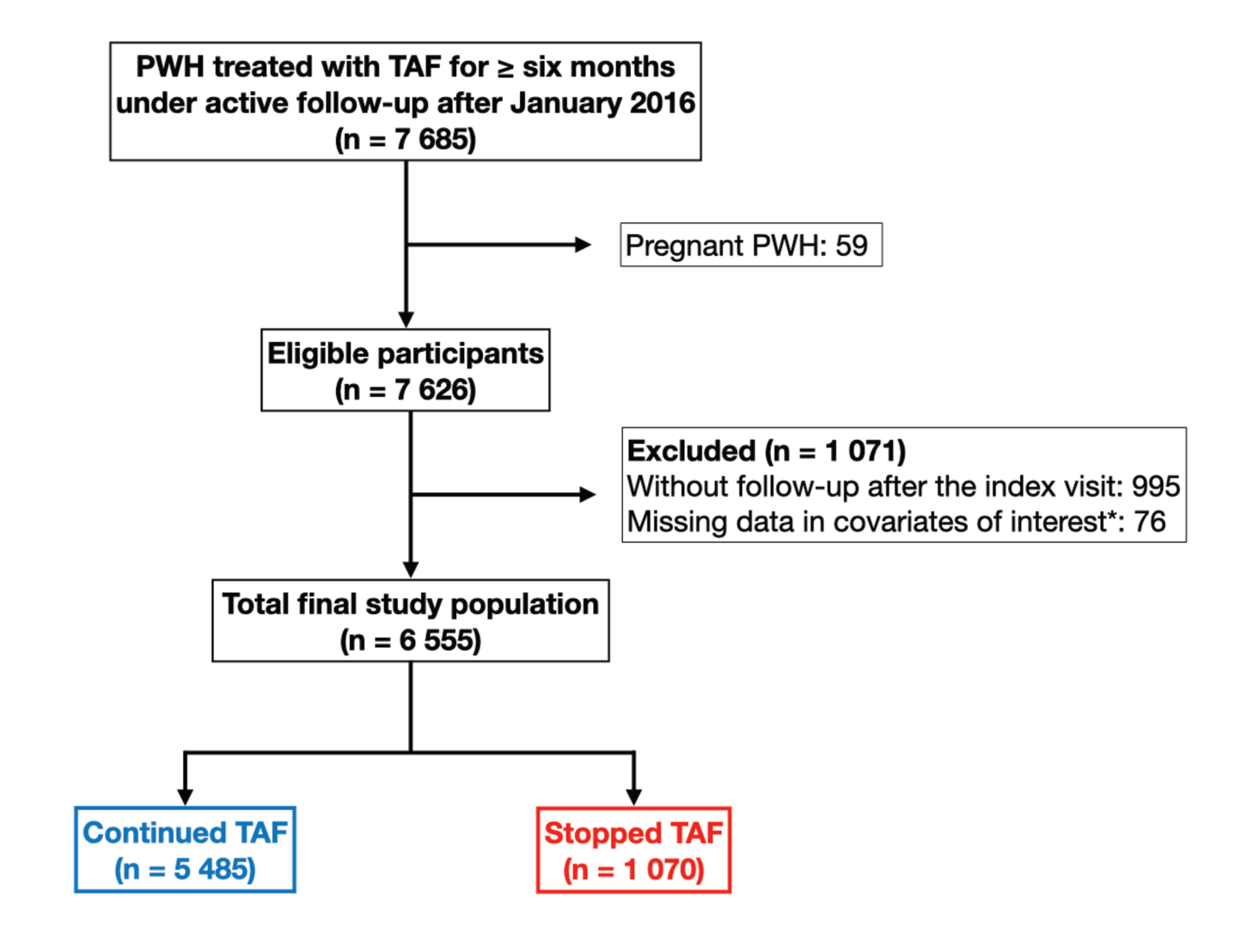
**

PWH = people with HIV; TAF = tenofovir alafenamide

*Covariables are baseline weight, baseline HIV-1 viral load, CD4-cell count, and smoking status.

***Figure S2.*** Changes in weight over time after index visit among overall population (superior panel) and stratified by sex (inferior panel).

**
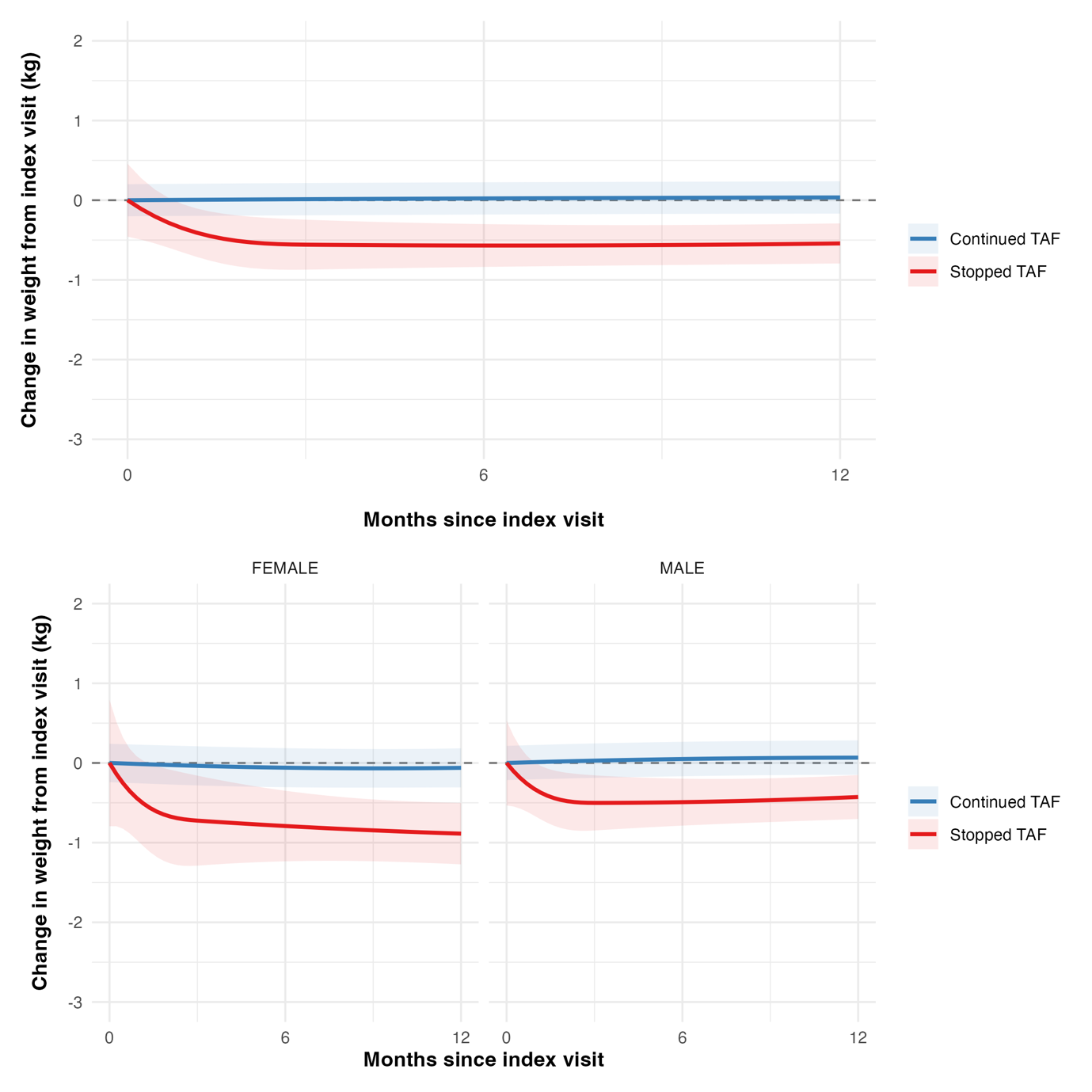
**

Mean changes in weight (line) and corresponding 95% CIs (shaded area) after discontinuing TAF compared to continuing TAF-based ART. Models are adjusted for age, sex, ethnicity, CD4-cell count, ART containing INSTI, physical activity, smoking status, and use of weight-modifying drugs. The model includes random intercepts for each individual. A total of 6’282 PWH were included in the analyses.

CIs = confidence intervals; TAF = tenofovir alafenamide; ART = antiretroviral therapy; INSTI = integrase strand transfer inhibitor

***Figure S3.*** Adjusted mean changes (95 CIs) in lipid levels from the index visit to 12 months thereafter.

**
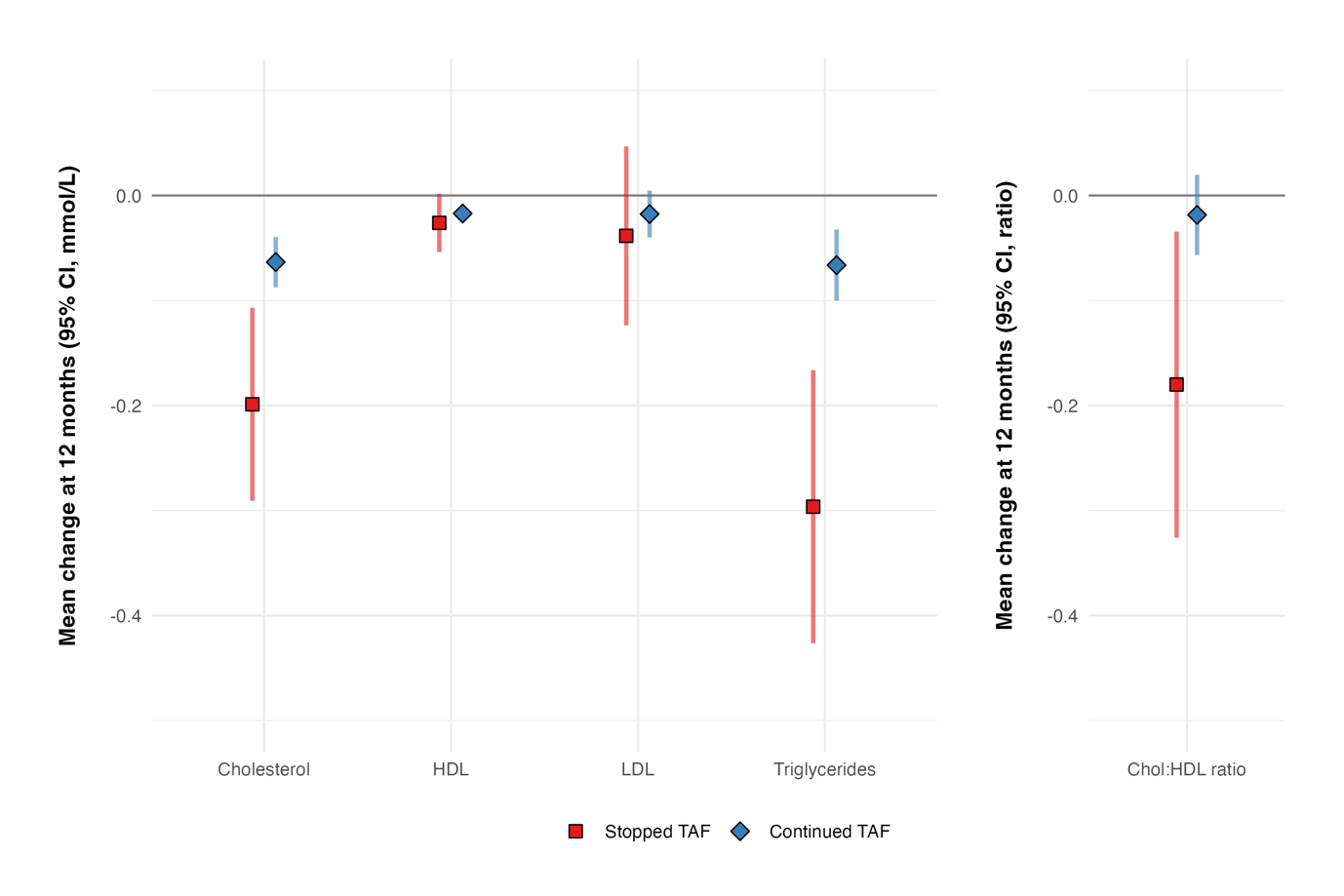
**

Mean changes (squares and diamonds) and 95% CIs (vertical line) in blood lipid values from the index visit to 12 months thereafter. Models were adjusted for age, sex, ethnicity, individual lipid level at baseline, and time-varying physical activity, weight, and use of lipid-lowering drugs. The model includes random intercepts for each individual. HDL = high-density lipoprotein; LDL = low density lipoprotein; TAF = tenofovir alafenamide.

***Figure S4.*** Changes in waist-to-hip ratio overtime, overall (superior panel) and stratified by sex (inferior panel).


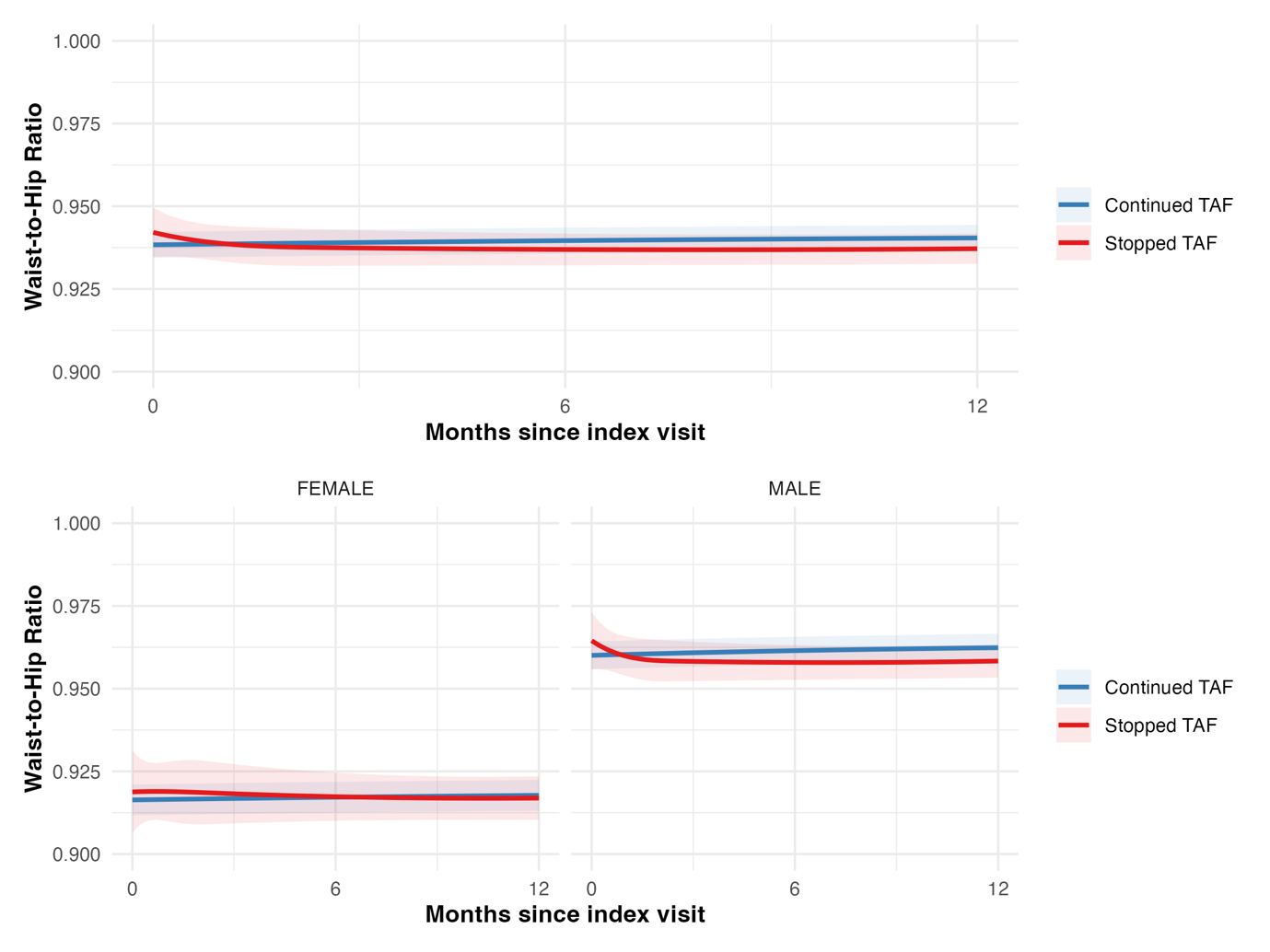
 Mean changes in waist-to-hip ratio (line) and corresponding 95% CIs (shaded area) after discontinuing TAF compared to individuals who continued TAF-based ART. Models were adjusted for age, sex, ethnicity, CD4-cell count, ART containing INSTI, physical activity, smoking status and use of weight-modifying drugs. The model includes random intercepts for each individual. A total of 6235 PWH were included in analyses.

CIs = confidence intervals; TAF = tenofovir alafenamide.

***Table S1.*** Adjusted mean differences in lipid levels after 12 months between individuals who continued TAF and those who switched from TAF to TDF.

| **Variable** | **Continued TAF, (95% CI)** | **Switched to TDF, (95% CI)** | **Difference between the two groups** | **p-value for difference** |
| --- | --- | --- | --- | --- |
| **Total cholesterol** | | | | < 0.001 |
| mmol/L | -0.06 (-0.09 to -0.04) | -0.44 (-0.65 to -0.24) | -0.38 (-0.59 to -0.17) |  |
| mg/dL | -2.5 (-3.4 to -1.5) | -17.1 (-25.1 to -9.2) | -14.6 (-22.7 to -6.6) |  |
| **HDL cholesterol** | | | | 0.543 |
| mmol/L | -0.02 (-0.02 to -0.01) | -0.09 (-0.15 to -0.03) | -0.07 (-0.14 to -0.01) |  |
| mg/dL | -0.07 (-0.9 to -0.4) | -3.5 (-6.0 to -1.1) | -2.9 (-5.3 to -0.4) | 0.020 |
| **LDL cholesterol** | | | | 0.075 |
| mmol/L | -0.02 (-0.04 to 0.001) | -0.19 (-0.38 to 0.00) | -0.18 (-0.37 to 0.02) |  |
| mg/dL | -0.7 (-1.5 to 0.2) | -7.4 (-14.9 to 0.0) | -6.8 (-14.3 to 0.7) |  |
| **Triglycerides** | | | | 0.032 |
| mmol/L | -0.07 (-0.10 to -0.03) | -0.38 (-0.66 to -0.10) | -0.31 (-0.59 to -0.03) |  |
| mg/dL | -5.9 (-8.9 to -2.9) | -33.3 (-58.1 to -8.5) | -27.3 (-52.2 to -2.3) |  |
| **Total cholesterol to HDL ratio** | | | |  |
|  | -0.02 (-0.06 to 0.02) | -0.14 (-0.47 to 0.19) | -0.13 (-0.46 to 0.21) | 0.456 |

TAF = tenofovir alafenamide; TDF = tenofovir disproxil fumarate; CI = confidence interval; HDL = high-density lipoprotein; IQR = interquartile range; LDL = low-density lipoprotein

Models adjusted for time fixed covariables age, sex, ethnicity, individual lipid level at baseline, and time-varying physical activity, weight, and use of lipid-lowering drugs.

***Table S2.*** Adjusted mean differences in lipid levels after 12 months between individuals who continued TAF and those who switched from TAF to DTG/3TC.

| **Variable** | **Continued TAF, (95% CI)** | **Switched to DTG/3TC, (95% CI)** | **Difference between the two groups** | **p-value for difference** |
| --- | --- | --- | --- | --- |
| **Total cholesterol** | | | | <0.001 |
| mmol/L | -0.06 (-0.09 to -0.04) | -0.29 (-0.43 to -0.16) | -0.23 (-0.37 to -0.09) |  |
| mg/dL | -2.5 (-3.4 to -1.5) | -11.3 (-16.4 to -6.1) | -8.9 (-14.1 to -3.6) |  |
| **HDL cholesterol** | | | | 0.02 |
| mmol/L | -0.02 (-0.02 to -0.01) | -0.09 (-0.15 to -0.03) | -0.07 (-0.14 to -0.01) |  |
| mg/dL | -0.07 (-0.9 to -0.4) | -3.5 (-6.0 to -1.1) | -2.9 (-5.3 to -0.4) |  |
| **LDL cholesterol** | | | | 0.79 |
| mmol/L | -0.02 (-0.04 to 0.001) | -0.03 (-0.16 to 0.09) | -0.02 (-0.14 to 0.11) |  |
| mg/dL | -0.7 (-1.5 to 0.2) | -1.3 (-6.1 to 3.5) | -0.7 (-5.6 to 4.2) |  |
| **Triglycerides** | | | | <0.001 |
| mmol/L | -0.07 (-0.10 to -0.03) | -0.51 (-0.69 to -0.32) | -0.44 (-0.63 to -0.25) |  |
| mg/dL | -5.9 (-8.9 to -2.9) | -44.7 (-61.3 to -28.2) | -39.0 (-55.8 to -22.1) |  |
| **Total cholesterol to HDL ratio** | | | | 0.005 |
|  | -0.02 (-0.06 to 0.02) | -0.33 (-0.54 to -0.12) | -0.31 (-0.53 to -0.1) |  |

TAF = tenofovir alafenamide; DTG = dolutegravir; 3TC = lamivudine; CI = confidence interval; HDL = high-density lipoprotein; IQR = interquartile range; LDL = low-density lipoprotein

Models adjusted for time fixed covariables age, sex, ethnicity, individual lipid level at baseline, and time-varying physical activity, weight, and use of lipid-lowering drugs.

***Table S3.*** Adjusted mean differences in lipid levels after 12 months between individuals who continued TAF and those who switched from TAF to CAB/RPV.

| **Variable** | **Continued TAF, (95% CI)** | **Switched to CAB/RPV, (95% CI)** | **Difference between the two groups** | **p-value for difference** |
| --- | --- | --- | --- | --- |
| **Total cholesterol** | | | |  |
| mmol/L | -0.06 (-0.09 to -0.04) | -0.29 (-0.43 to -0.16) | -0.23 (-0.37 to -0.09) | <0.001 |
| mg/dL | -2.5 (-3.4 to -1.5) | -11.3 (-16.4 to -6.1) | -8.9 (-14.1 to -3.6) |  |
| **HDL cholesterol** | | | | 0.206 |
| mmol/L | -0.02 (-0.02 to -0.01) | 0.16 (-0.18 to 0.50) | 0.22 (-0.12 to 0.56) |  |
| mg/dL | -0.07 (-0.9 to -0.4) | 6.1 (-7.1 to 19.3) | 8.5 (-4.7 to 21.7) |  |
| **LDL cholesterol** | | | | 0.311 |
| mmol/L | -0.02 (-0.04 to 0.001) | 0.15 (-0.17 to 0.47) | 0.17 (-0.16 to 0.49) |  |
| mg/dL | -0.7 (-1.5 to 0.2) | 5.8 (-6.6 to 18.3) | 6.5 (-6.0 to 19.0) |  |
| **Triglycerides** | | | | 0.72 |
| mmol/L | -0.07 (-0.10 to -0.03) | 0.02 (-0.44 to 0.48) | 0.08 (-0.38 to 0.55) |  |
| mg/dL | -5.9 (-8.9 to -2.9) | 1.6 (-39.3 to 42.6) | 7.5 (-33.5 to 48.6) |  |
| **Total cholesterol to HDL ratio** | | | | 0.814 |
|  | -0.02 (-0.06 to 0.02) | 0.05 (-0.50 to 0.60) | 0.07 (-0.49 to 0.62) |  |

TAF = tenofovir alafenamide; CAB = cabotegravir; RPV = rilpivirine; CI = confidence interval; HDL = high-density lipoprotein; IQR = interquartile range; LDL = low-density lipoprotein

Models adjusted for time fixed covariables age, sex, ethnicity, individual lipid level at baseline: and time-varying physical activity, weight, and use of lipid-lowering drugs.

***Table S4.*** Adjusted mean differences in lipid levels after 12 months between individuals who continued TAF and those who switched from TAF to other ART.

| **Variable** | **Continued TAF, (95% CI)** | **Switched to other ART, (95% CI)** | **Difference between the two groups** | **p-value for difference** |
| --- | --- | --- | --- | --- |
| **Total cholesterol** | | | | 0.234\| |
| mmol/L | -0.06 (-0.09 to -0.04) | 0.05 (-0.14 to 0.24) | 0.11 (-0.07 to 0.3) |  |
| mg/dL | -2.5 (-3.4 to -1.5) | 2.0 (-5.3 to 9.3) | 4.4 (-2.9 to 11.8) |  |
| **HDL cholesterol** | | | | 0.144 |
| mmol/L | -0.02 (-0.02 to -0.01) | 0.02 (-0.03 to 0.08) | 0.04 (-0.01 to 0.1) |  |
| mg/dL | -0.07 (-0.9 to -0.4) | 1.0 (-1.2 to 3.2) | 1.6 (-0.6 to 3.9) |  |
| **LDL cholesterol** | | | | 0.827 |
| mmol/L | -0.02 (-0.04 to 0.001) | 0.00 (-0.17 to 0.18) | 0.02 (-0.16 to 0.2) |  |
| mg/dL | -0.7 (-1.5 to 0.2) | 0.1 (-6.6 to 6.9) | 0.8 (-6.1 to 7.6) |  |
| **Triglycerides** | | | | 0.367\| |
| mmol/L | -0.07 (-0.10 to -0.03) | 0.05 (-0.2 to 0.31) | 0.12 (-0.14 to 0.38) |  |
| mg/dL | -5.9 (-8.9 to -2.9) | 4.8 (-18.2 to 27.7) | 10.6 (-12.5 to 33.8) |  |
| **Total cholesterol to HDL ratio** | | | |  |
|  | -0.02 (-0.06 to 0.02) | -0.02 (-0.32 to 0.28) | 0 (-0.31 to 0.3) | 0.992 |

TAF = tenofovir alafenamide; CI = confidence interval; HDL = high-density lipoprotein; IQR = interquartile range; LDL = low-density lipoprotein

Models adjusted for time fixed covariables age, sex, ethnicity, individual lipid level at baseline, and time-varying physical activity, weight, and use of lipid-lowering drugs.
